# Salvage Posterior C1–C2 Fusion for Odontoid Nonunion After Failed Nonoperative Management: A Propensity Score-Matched Comparison with Primary Fusion

**DOI:** 10.3390/jcm15103887

**Published:** 2026-05-18

**Authors:** Sapan Patel, Hershil A. Patel, Rohan I. Suresh, Jake Carbone, Gerald Kidd, Abel K. Lindley, Ethan Yang, Antoan Koshar, Ryan Curto, Husni Alasadi, Usman Zareef, Evan Honig, Alexander Padovano, Louis Bivona, Daniel Cavanaugh, Eugene Koh, Steven C. Ludwig, Julio J. Jauregui

**Affiliations:** Division of Spine Surgery, Department of Orthopaedics, University of Maryland School of Medicine, Baltimore, MD 21201, USAhershilpatel@som.umaryland.edu (H.A.P.); jjcarbone20@gmail.com (J.C.); gkidd@som.umaryland.edu (G.K.); abel.lindley@som.umaryland.edu (A.K.L.); ethan.yang@som.umaryland.edu (E.Y.); antoan.koshar@som.umaryland.edu (A.K.); halasadi@som.umaryland.edu (H.A.); uzareef@som.umaryland.edu (U.Z.); ehonig@som.umaryland.edu (E.H.); alexander.padovano@som.umaryland.edu (A.P.); lbivona@som.umaryland.edu (L.B.); dcavanaugh@som.umaryland.edu (D.C.); ekoh@som.umaryland.edu (E.K.); jjauregui@som.umaryland.edu (J.J.J.)

**Keywords:** odontoid fracture, C1–C2 fusion, posterior cervical fusion, salvage surgery, nonoperative management, length of stay, cervical alignment, complications

## Abstract

**Background/Objectives**: Posterior C1–C2 fusion is commonly used for unstable traumatic odontoid injuries, but it is less commonly used for patients who initially undergo nonoperative management and later require salvage fusion. This study compared hospital length of stay, short-term complications, and postoperative radiographic alignment between salvage posterior C1–C2 fusion after failed nonoperative management and primary posterior C1–C2 fusion. **Materials and Methods**: A retrospective cohort study was performed of 106 adult patients who underwent posterior C1–C2 instrumented fusion for traumatic cervical spine injuries from 2011 to 2023. Patients were stratified into the salvage fusion group after radiographic nonunion following attempted nonoperative management with external immobilization or the primary fusion group, who underwent initial surgical management. The primary outcome was hospital length of stay. Secondary outcomes included postoperative radiographic alignment, screw loosening, hardware failure, revision surgery, and 30-day emergency department visits. Propensity score matching and full-cohort augmented inverse probability weighting were used to account for baseline differences between groups. **Results**: Twenty-seven patients underwent salvage fusion and 79 underwent primary fusion. Propensity score matching produced 25 matched pairs. In the matched cohort, salvage fusion was associated with significantly shorter length of stay than primary fusion, with a median of 2 versus 5 days, respectively (*p* < 0.001). This remained significant in the full-cohort augmented inverse probability weighting analysis, where salvage fusion was associated with a 2.41-day reduction in length of stay (95% CI, −3.63 to −1.19; *p* < 0.001). Short-term complications were uncommon in both groups, and no clear sign of increased screw loosening, hardware failure, revision surgery, or 30-day emergency department visits was observed in the salvage cohort. Salvage fusion was also associated with lower postoperative C2–C7 lordosis and a greater C1 lamina–occiput distance. **Conclusions**: Salvage posterior C1–C2 fusion for radiographic nonunion after attempted nonoperative management was not associated with higher short-term complication rates compared with primary fusion. While surgical-admission length of stay was shorter in the salvage cohort, this difference should be interpreted cautiously because salvage and primary fusion occur in different admission contexts and do not reflect the total episode-of-care burden. Early postoperative alignment differences were observed, but these were not correlated with clinical outcomes or longitudinal imaging, and their long-term significance remains unclear. Future multicenter studies should evaluate total healthcare utilization, fusion status, longitudinal alignment, and patient-reported outcomes after salvage C1–C2 fusion.

## 1. Introduction

Odontoid fractures are common upper cervical spine injuries in adults, accounting for approximately 9% to 15% of adult cervical spine fractures, with type II fractures comprising the majority of odontoid injuries [1]. These injuries are particularly consequential in older adults, where treatment decisions must balance fracture stability, union potential, neurologic risk, medical frailty, and the morbidity of prolonged immobilization or surgery [2]. Surgical stabilization is generally associated with higher fusion rates, whereas nonoperative management may avoid perioperative morbidity and still provide acceptable clinical outcomes in selected patients [3]. This tension has made the management of type II odontoid fractures persistently controversial, particularly in elderly or medically complex patients [4].

Most existing studies compare operative and nonoperative treatment as competing upfront strategies, but this binary framework does not fully address patients who initially undergo external immobilization and later require posterior C1–C2 fusion after failed conservative management [3]. This salvage pathway is clinically important because nonoperative treatment can result in high radiographic nonunion rates, yet nonunion does not always translate into poor function or the need for surgery [5,6]. Fibrous nonunion may be acceptable in carefully selected patients without dynamic instability, neurologic deficit, or high risk of reinjury, but failed nonoperative management raises a separate question regarding the safety and perioperative burden of delayed fusion [7]. Therefore, it remains unclear whether salvage posterior C1–C2 fusion after failed nonoperative management carries the risk of longer hospitalization periods and complications, or different postoperative alignment compared with primary fusion performed during the acute treatment pathway.

The purpose of this study was to compare patients undergoing salvage posterior C1–C2 fusion for radiographic nonunion after attempted nonoperative management with those undergoing primary posterior C1–C2 fusion for traumatic cervical spine injury. Specifically, we aimed to (1) compare the length of stay for the hospitalization during which fusion was performed; (2) evaluate short-term postoperative complications, including screw loosening, hardware failure, revision surgery, and 30-day emergency department visits; and (3) compare immediate postoperative radiographic alignment between treatment pathways. Using propensity score matching and augmented inverse probability weighting in a cohort of 106 patients, we sought to reduce measured baseline imbalance and characterize the early perioperative and radiographic profile of salvage fusion relative to primary fusion.

## 2. Materials and Methods

### 2.1. Study Design

We performed a retrospective cohort study of all adult patients who underwent posterior C1–C2 instrumented fusion for traumatic cervical spine injuries at a single academic medical center between 2011 and 2023. The study was conducted under institutional review board approval, and the need for informed consent was waived, given its retrospective design.

### 2.2. Patient Population and Selection Criteria

Patients were eligible for inclusion if they were 18 years of age or older, underwent posterior C1–C2 instrumented fusion with bilateral C1 lateral mass and C2 screw fixation, and had a traumatic cervical spine injury as the primary surgical indication. Patients were excluded if the indication for fusion was primarily degenerative, inflammatory, oncologic, or congenital. All odontoid fracture types were included. No minimum follow-up threshold was applied. A total of 106 patients met the inclusion criteria.

### 2.3. Treatment Pathway Definition

Patients were stratified by treatment pathway into two groups. The salvage group comprised patients who initially underwent a trial of nonoperative management with external immobilization (rigid cervical collar or halo vest) for their traumatic odontoid injury, subsequently failed this nonoperative strategy, and were then taken for posterior C1–C2 fusion for radiographic nonunion. The primary group comprised patients who underwent posterior C1–C2 fusion as initial surgical management of their acute traumatic injury without a preceding trial of nonoperative management. Treatment pathway assignment was determined by the initial management decision and documented in the electronic medical record.

### 2.4. Surgical Technique

All procedures were performed through a standard posterior midline approach with bilateral C1 lateral mass and C2 instrumentation using either pedicle or pars (isthmus) screws. The C2 screw type was selected at the attending surgeon’s discretion based on preoperative computed tomography assessment of pedicle anatomy, vertebral artery course, and perceived risk of vascular injury. Four surgeons contributed cases to the cohort. Bone graft was used in 96.2% of cases.

### 2.5. Radiographic Measurement

Immediate postoperative plain radiographs obtained within 24 h of surgery served as the primary radiographic data source. We measured C2 slope, C2–3 segmental lordosis, C0–2 Cobb angle, C2–7 lordosis, C2 sagittal vertical axis (SVA), C1 lamina–occiput distance, and C2–3 listhesis. All measurements were performed by a single trained reviewer blinded to clinical outcomes. Formal interobserver and intraobserver reliability testing was not performed. Radiographic nonunion was determined from available follow-up radiographs and/or computed tomography reports, based on documented absence of osseous union or a persistent fracture line at the odontoid fracture site.

### 2.6. Outcomes

The primary outcome was hospital length of stay (LOS) in days. Secondary outcomes included postoperative radiographic alignment parameters, screw loosening, revision spine surgery, hardware failure, and 30-day emergency department visits. Complications were ascertained through review of operative reports, discharge summaries, and all available outpatient follow-up documentation.

### 2.7. Comorbidity and Injury Classification

The Charlson Comorbidity Index (CCI) was calculated using the original 1987 Charlson weights without age adjustment [8]. Injury mechanism was classified as high energy (motor vehicle collision, fall from height greater than standing height, or direct high-velocity impact) or low energy.

### 2.8. Statistical Analysis

Continuous variables are reported as the median with interquartile range (IQR). Categorical variables are reported as the count and percentage. Unadjusted between-group comparisons used the Mann–Whitney U test or Fisher’s exact test as appropriate.

To reduce the measured baseline imbalance between the nonrandomized treatment groups, we performed propensity score matching. The propensity score was estimated via multivariable logistic regression using age, sex, body mass index (BMI), CCI, Type II odontoid fracture, and high-energy mechanisms as covariates. Covariates were selected based on clinical relevance and availability across the full cohort. Variables such as fracture displacement, dynamic stability, neurologic status, bone quality, frailty measures beyond CCI, immobilization-specific details, and time from injury to surgery were not uniformly available and, therefore, were not included in the propensity model. One-to-one nearest-neighbor matching without replacement was performed on the logit of the propensity score with a caliper of 0.2 standard deviations of the logit propensity score [9]. Covariate balance before and after matching was assessed using standardized mean differences (SMDs), with |SMD| below 0.2 considered an acceptable balance. In the matched cohort, continuous outcomes were compared using the Mann–Whitney U test and binary outcomes were assessed using the McNemar test for paired data.

To augment the matched analysis and utilize the full cohort, we additionally estimated average treatment effects (ATEs) using augmented inverse probability weighting (AIPW): a doubly robust estimator that combines stabilized inverse probability of treatment weighting with outcome regression [10]. AIPW remains consistent if either the propensity score model or the outcome regression model is correctly specified. Stabilized weights were capped at the 99th percentile to limit the influence of extreme weights. Variance was estimated from the empirical variance in the influence function, and 95% confidence intervals were constructed using Wald-type limits.

To evaluate the robustness of the primary LOS finding to unmeasured confounding, we calculated the E-value using the approach of VanderWeele and Ding [11]. The E-value represents the minimum strength of association, measured on the risk ratio scale, that an unmeasured confounder would need to have with both treatment assignment and outcome, above and beyond the measured covariates, to fully explain the observed association. For the continuous LOS outcome, the observed effect was converted to an approximate risk ratio via the relation RR = exp(0.91 × Cohen’s d).

Given the low expected frequency of postoperative complications, complication endpoints were analyzed descriptively using absolute event rates and risk differences with 95% confidence intervals. These analyses were intended to identify whether there was a clear signal of increased short-term complication risk in the salvage cohort rather than to establish definitive non-inferiority for rare adverse events. Statistical significance was defined as a two-sided *p*-value below 0.05 for tests of superiority. Because this was a retrospective cohort of all eligible patients who underwent posterior C1–C2 fusion during the study period, no a priori sample size calculation was performed. Analyses were conducted using available data without imputation. For outcomes or radiographic parameters with incomplete data, analyses were performed using available observations. All analyses were performed using Python version 3.12 (SciPy, NumPy).

## 3. Results

### 3.1. Population Characteristics

During the study period, 106 patients met the inclusion criteria. The cohort had a median age of 72 years (IQR 64–79) and 52.8% were male (56/106). The median BMI was 25 kg/m^2^, and the median CCI was 0. Type II odontoid fractures accounted for 83.0% of injuries (88/106), and type III fractures accounted for 14.2% (15/106). High-energy mechanism was documented in 33.0% of cases (35/106). Sixty-five patients (61.3%) received C2 pedicle screws and 41 (38.7%) received C2 pars screws. The median follow-up was 197 days (IQR 71–396).

Seventy-nine patients (74.5%) underwent primary C1–C2 fusion as a form of initial surgical management. Twenty-seven patients (25.5%) underwent salvage fusion after radiographic nonunion following attempted nonoperative management. All patients in the salvage cohort had radiographic nonunion documented before delayed posterior C1–C2 fusion. Baseline characteristics of the unmatched cohorts are presented in Table 1. Salvage patients were younger than primary patients (median 69 vs. 73 years, *p* = 0.060) and had a modestly higher prevalence of high-energy mechanisms and CCI (Table 1).

### 3.2. Propensity Score Matching

**Figure 1:**
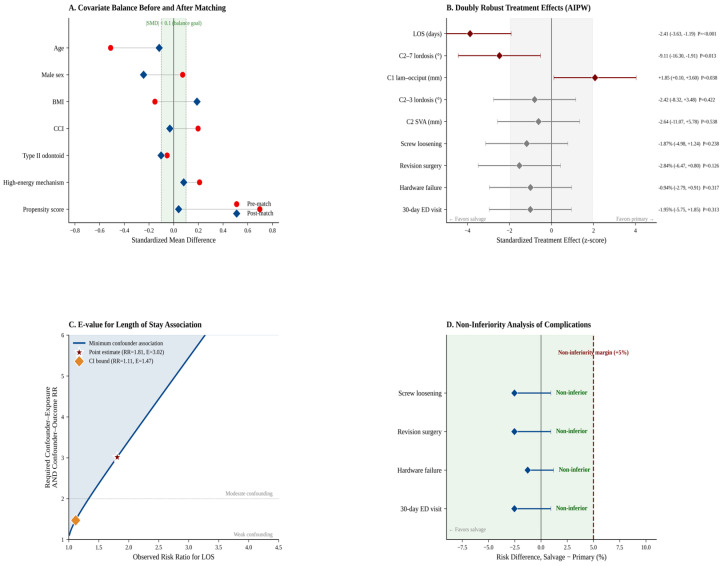
Integrated statistical analysis of salvage versus primary C1–C2 fusion. (**A**) Love plot of standardized mean differences (SMDs) for each covariate before (red circles) and after (blue diamonds) 1:1 propensity score matching. The green band indicates the balance goal (|SMD| < 0.10). Propensity score imbalance decreased from SMD +0.70 pre-match to +0.04 post-match. (**B**) Doubly robust average treatment effects (AIPW) across all outcomes, displayed on a standardized z-score scale with corresponding point estimates and 95% confidence intervals on the right. The gray shaded band spans z = −1.96 to +1.96 and represents the non-significant zone; effect estimates whose confidence interval crosses this band are not statistically significant at α = 0.05. Dark-red markers indicate statistical significance (*p* < 0.05). Salvage fusion was associated with significantly shorter LOS, lower postoperative C2–7 lordosis, and greater C1 lamina-occiput distance. (**C**) E-value analysis for the length of stay finding. The solid blue curve plots the minimum confounder–exposure and confounder–outcome association (on the risk ratio scale, assumed symmetric) required to fully explain away an observed risk ratio; the blue shaded area above the curve represents the parameter space in which unmeasured confounding would be sufficient to nullify the observed effect. The point estimate E-value of 3.02 (red star) indicates that unmeasured confounding would need to be substantial to nullify the observed association; the CI-bound E-value of 1.47 (orange diamond) reflects moderate robustness to residual confounding. (**D**) Non-inferiority analysis of complication endpoints, with the pre-specified +5% margin shown as a dashed red line. The green shaded area to the left of this margin represents the non-inferiority zone; if the upper bound of the 95% confidence interval for the risk difference falls within this green region, salvage fusion is concluded non-inferior to primary fusion for that endpoint. All four complication outcomes met the non-inferiority criterion. AIPW = augmented inverse probability weighting; ATE = average treatment effect; CI = confidence interval; ED = emergency department; LOS = length of stay.

The propensity score for salvage fusion was estimated using age, sex, BMI, CCI, type II fracture, and high-energy mechanisms. The median propensity score was 0.293 (IQR 0.205–0.438) in salvage patients and 0.211 (IQR 0.151–0.251) in primary patients. One-to-one nearest-neighbor matching with a caliper of 0.143 on the logit propensity score produced 25 matched pairs, with a median match distance of 0.022 on the logit scale. Covariate balance improved substantially after matching, with the propensity score SMD decreasing from +0.70 to +0.04 (Figure 1A, Table 2). Age, CCI, and high-energy mechanism achieved |SMD| below 0.15 post-match. A residual imbalance in male sex (a post-match SMD of −0.24) was observed and is addressed in the limitations.

### 3.3. Length of Stay (Primary Outcome)

The primary outcome of hospital length of stay differed significantly between groups. In the matched cohort, salvage patients had a median LOS of 2 days (IQR 2–3) compared with 5 days (IQR 3–6) in primary patients (*p* < 0.001, Table 3, Figure 1B). Using the full cohort with augmented inverse probability weighting, the doubly robust average treatment effect of salvage treatment on LOS was −2.41 days (95% CI −3.63 to −1.19, *p* < 0.001, Table 4). The corresponding standardized effect size (Cohen’s d) was 0.65, consistent with a medium-to-large effect. The E-value for unmeasured confounding was 3.02 for the point estimate and 1.47 for the 95% confidence interval bound closest to the null (Table 5, Figure 1C), indicating that an unmeasured confounder would need to be associated with both treatment assignment and LOS by a risk ratio of at least 3.02 to fully explain away the observed association.

### 3.4. Complications

Postoperative complications were uncommon in both groups. Screw loosening occurred in two patients, hardware failure occurred in one patient, revision spine surgery occurred in two patients, and 30-day emergency department visits were needed for two patients. In the matched cohort, no complications occurred in the salvage group, while complications in the primary group remained rare (Table 3). Given the low number of events, these findings should be interpreted descriptively. Overall, there was no clear signal of increased short-term screw loosening, hardware failure, revision surgery, or 30-day emergency department visits among salvage patients. (Table 6, Figure 1D).

### 3.5. Postoperative Radiographic Alignment

Immediate postoperative radiographic alignment differed between groups. In the AIPW analysis of the full cohort, salvage patients demonstrated significantly lower C2–7 lordosis than primary patients (ATE −9.11°, 95% CI −16.30 to −1.91, *p* = 0.013) and a significantly greater C1 lamina–occiput distance (ATE +1.85 mm, 95% CI +0.10 to +3.60, *p* = 0.038, Table 4). The differences in C2–3 segmental lordosis (ATE −2.42°, *p* = 0.422) and C2 SVA (ATE −2.65 mm, *p* = 0.538) were not statistically significant. In the matched cohort, C2–7 lordosis trended lower in salvage patients (20.8° vs. 33.6°, *p* = 0.101) but did not reach conventional statistical significance, likely reflecting reduced power from the 1:1 matched sample (Table 3).

### 3.6. Summary of Findings

Taken across the matched and doubly robust analyses, salvage C1–C2 fusion was associated with shorter LOS for the fusion hospitalization and showed no clear signal of increased short-term complications compared with primary fusion. Immediate postoperative radiographic differences were also observed, including lower C2–C7 lordosis and greater C1 lamina–occiput distance, although their clinical significance remains uncertain.

## 4. Discussion

In the present study, salvage posterior C1–C2 fusion for radiographic nonunion after attempted nonoperative management was associated with shorter LOS for hospitalization when salvage fusion was performed compared with primary fusion. In the matched analysis, salvage patients had a median LOS of 2 days compared with 5 days in the primary fusion cohort, and this finding remained consistent in the full-cohort AIPW analysis. However, this finding should be interpreted cautiously because the two treatment pathways likely differed in the admission context. Primary fusion often occurs during acute trauma admission, where LOS may be influenced by trauma workup, medical optimization, associated injuries, inpatient disposition, and discharge planning. In contrast, salvage fusion occurs after delayed recognition of nonunion, which may allow for more controlled admission timing, preoperative planning, and discharge coordination. Therefore, the observed LOS difference should not be interpreted as evidence that salvage fusion reduces total healthcare utilization or the overall burden of the fracture episode. The LOS observed in our primary fusion cohort is shorter than typical reports in contemporary posterior C1–C2 fusion in the literature, where elderly surgical cohorts have commonly reported hospital stays of approximately 9 to 14 days, with the longest stays in octogenarian and nonagenarian series [3,12,13,14]. In this context, the shorter LOS observed in the salvage cohort should not be interpreted as proof that delayed surgery reduces the total burden of care across the entire fracture episode. Rather, it suggests that when patients initially managed nonoperatively ultimately require posterior C1–C2 fusion, salvage surgery does not impose an added hospitalization penalty compared with primary fusion. Unmeasured factors related to admission context, surgical timing, discharge planning, and institutional care pathways may still contribute to this difference, but the consistency of the matched and doubly robust analyses supports the clinical interpretation that failed nonoperative management does not necessarily translate into a more burdensome surgical admission. Rather, these findings suggest that among patients who ultimately require posterior C1–C2 fusion for radiographic nonunion after attempted immobilization, delayed salvage surgery does not appear to impose an added surgical-admission hospitalization penalty compared with primary fusion.

Beyond LOS, a central concern with delayed fusion after nonoperative treatment failure is whether a salvage pathway introduces additional perioperative or hardware-related risk. In the present study, short-term complications were uncommon, and no clear signs of increased screw loosening, hardware failure, revision surgery, or 30-day emergency department visits were observed among salvage patients. This finding is reassuring, but it should not be interpreted as definitive evidence of non-inferiority because the absolute number of complications was low and the study was underpowered to detect rare but clinically important adverse events. This finding is clinically relevant because posterior C1–C2 fusion is generally considered a reliable stabilization strategy for unstable or nonunited odontoid fractures; however, published geriatric odontoid series demonstrate that surgical treatment can carry meaningful morbidity, particularly from medical complications rather than construct-specific failure [12,15,16]. In a systematic review of older adults with type II odontoid fractures, surgical treatment was associated with higher fusion rates but also higher overall complication rates than nonoperative management, reinforcing the fact that the benefit of stabilization must be balanced against perioperative risk [3]. More recent prospective comparative data similarly showed that secondary treatment occurred less often after surgery than conservative treatment, but surgically treated patients still required secondary procedures for issues such as persistent instability, nonunion, hardware removal, or wound revision [17]. Against this backdrop, the absence of increased hardware-related complications or revision surgery in the salvage cohort suggests that radiographic nonunion after attempted immobilization did not translate into a clearly more hazardous surgical episode once posterior fusion was performed. However, these findings should be interpreted cautiously because the absolute number of complications was low, and the study was not powered to detect rare but clinically important events such as vertebral artery injury, neurologic deterioration, deep infection, or mortality. Therefore, the most appropriate interpretation is not that salvage fusion is risk-free, but rather that delayed posterior C1–C2 fusion for radiographic nonunion after attempted immobilization did not show a clear signal of increased short-term complications compared with upfront fusion in this cohort. These findings should, therefore, be interpreted as an early perioperative safety comparison rather than a definitive assessment of long-term clinical effectiveness. In particular, CT-confirmed fusion status, pain relief, functional recovery, mortality, and quality-of-life outcomes were not uniformly available, limiting our ability to determine whether salvage fusion achieves equivalent durable clinical success compared with primary fusion.

Although salvage fusion did not demonstrate increased risk for short-term complications, the third aim of this study was to identify differences in immediate postoperative radiographic alignment between treatment pathways. In the full-cohort AIPW analysis, salvage fusion was associated with lower postoperative C2–C7 lordosis and greater C1 lamina–occiput distance compared with primary fusion, while C2–3 segmental lordosis and C2 SVA did not significantly differ between groups. These findings are relevant because upper cervical fixation can influence subaxial cervical alignment through reciprocal compensatory changes, and prior radiographic studies have shown interdependence between upper cervical alignment, C1–C2 fusion angle, and C2–C7 alignment [18,19,20]. Postoperative subaxial kyphotic change after upper cervical fixation has also been described, supporting the concept that fixation at C1–C2 may alter downstream cervical alignment even when the construct itself remains mechanically stable [21]. In the salvage setting, delayed fixation after a period of nonoperative management may also permit chronic remodeling at the fracture site, altered reducibility, or compensatory upper cervical and subaxial alignment changes before fusion, which could contribute to differences in immediate postoperative alignment. In the broader posterior cervical fusion literature, positive C2–C7 sagittal malalignment has been associated with greater disability, underscoring why postoperative sagittal parameters warrant attention even when early hardware complications are uncommon [22]. However, the clinical significance of the alignment differences observed in the present study remains uncertain because these measurements were obtained from immediate postoperative radiographs and were not directly linked to pain, neurologic recovery, patient-reported outcomes, CT-confirmed fusion status, or longitudinal alignment. Therefore, these findings should be interpreted as early radiographic differences of uncertain clinical significance rather than evidence of clinically meaningful malalignment. Therefore, these radiographic findings should be interpreted as hypothesis-generating rather than evidence of clinically meaningful malalignment. Taken together, salvage fusion appears to avoid the added burden of short-term complications but may be associated with subtle postoperative alignment differences that merit further study with longer-term radiographic follow-up and patient-centered outcomes.

This study has several limitations. First, although propensity score matching and augmented inverse probability weighting were used to reduce imbalance across measured covariates, the retrospective design precludes causal inference and does not eliminate confounding by indication. Treatment pathway selection may have been influenced by clinically important variables that were not uniformly available for inclusion in the propensity model, including fracture displacement, dynamic stability, neurologic status, frailty beyond CCI, bone quality, immobilization type and compliance, time from injury to surgery, surgeon preference, and institutional care pathways. Therefore, the adjusted findings should be interpreted as associations rather than evidence of a causal effect of salvage versus primary fusion. Although all salvage patients underwent delayed fusion for radiographic nonunion, the retrospective design limited our ability to uniformly characterize the clinical context of nonunion, including symptom severity, degree of fracture displacement, dynamic instability, immobilization compliance, and surgeon-specific thresholds for proceeding with delayed fixation. Second, LOS was measured only for the hospitalization during which posterior C1–C2 fusion was performed rather than the entire fracture episode. This distinction is particularly important because primary fusion often occurs during admission for acute trauma, whereas salvage fusion occurs after delayed identification of nonunion and may have been performed in a more scheduled or controlled admission context. As a result, differences in admission pathway, trauma workup, preoperative planning, discharge disposition, and prior nonoperative treatment burden may have contributed to the shorter LOS observed in the salvage cohort. This finding should, therefore, not be interpreted as evidence of lower total healthcare utilization or lower overall treatment burden. Third, postoperative complications were rare, which limited the statistical power to detect meaningful differences between groups. Therefore, the absence of increased complications in the salvage cohort should not be interpreted as providing evidence of this method’s short-term safety, definitive evidence of non-inferiority, or equivalent long-term effectiveness. Rare but clinically important events such as vertebral artery injury, neurologic deterioration, deep infection, mortality, delayed hardware failure, pseudarthrosis, or late revision surgery may not be adequately captured in this sample. Additionally, longer-term clinical outcomes, including CT-confirmed fusion status, pain relief, functional recovery, quality of life, and mortality, were not uniformly available, limiting our ability to determine whether salvage fusion achieves equivalent durable clinical success compared with primary fusion. Similarly, radiographic alignment was assessed using immediate postoperative imaging only and was not correlated with longitudinal radiographic change, CT-confirmed fusion, pain, function, or quality-of-life outcomes; therefore, the clinical significance of these differences in alignment remains uncertain. Fourth, follow-up duration differed between groups, with longer follow-up needed in the salvage cohort. This may have influenced detection of delayed complications, including screw loosening, hardware failure, or revision surgery. Although time-to-event analysis would be ideal for delayed events, the very low number of complications in this cohort limits the reliability of formal survival modeling. Therefore, complications should be interpreted descriptively and primarily as signaling no clear short-term safety for this method rather than definitive equivalence of long-term complication risks. Larger multicenter studies with standardized follow-up are needed to validate these findings and clarify the long-term clinical significance of salvage posterior C1–C2 fusion after failed nonoperative management.

## 5. Conclusions

Salvage posterior C1–C2 fusion for radiographic nonunion after attempted nonoperative management represents an important but understudied pathway in the treatment of traumatic upper cervical spine injuries. In this propensity score-matched cohort, salvage fusion was not associated with higher short-term complication rates compared with primary fusion, suggesting that delayed posterior stabilization for nonunion does not necessarily translate into a higher-risk surgical episode. Although salvage fusion was associated with shorter LOS for the fusion hospitalization, this finding should be interpreted cautiously because LOS did not capture the total fracture episode or prior nonoperative treatment burden. Future multicenter studies with standardized longitudinal follow-up, CT-confirmed fusion assessment, total healthcare utilization, and patient-reported outcomes are needed to clarify the durability and clinical significance of these findings.

## Figures and Tables

**Table 1:** Unmatched baseline characteristics by treatment pathway.

Variable	Salvage (n = 27)	Primary (n = 79)	*p*-Value
Age (years), median (IQR)	69.0 (48.0–77.0)	73.0 (66.5–79.0)	0.060
Male sex, n (%)	15 (55.6%)	41 (51.9%)	0.916
BMI (kg/m^2^), median (IQR)	25.0 (22.5–26.0)	25.0 (22.0–29.0)	0.807
CCI, median (IQR)	0.0 (0.0–1.0)	0.0 (0.0–1.0)	0.460
High-energy mechanism, n (%)	11 (40.7%)	24 (30.4%)	0.403
Type II odontoid, n (%)	22 (81.5%)	66 (83.5%)	1.000
Type III odontoid, n (%)	4 (14.8%)	11 (13.9%)	1.000
C2 pedicle screw, n (%)	13 (48.1%)	52 (65.8%)	0.162
Bone graft use, n (%)	26 (96.3%)	76 (96.2%)	1.000
Length of stay (days), median (IQR)	2.0 (2.0–3.0)	5.0 (3.0–7.0)	<0.001
Follow-up (days), median (IQR)	354 (119–517)	184 (53–368)	0.061

Values are median (IQR) or n (%). *p*-values from the Mann–Whitney U test (continuous) or Fisher’s exact test (categorical). *p*-values indicate statistical significance (*p* < 0.05). Salvage = patients who underwent C1–C2 fusion after a failed trial of nonoperative management; primary = patients who underwent C1–C2 fusion as initial surgical management of the acute injury. BMI = body mass index; CCI = Charlson Comorbidity Index; IQR = interquartile range.

**Table 2:** Covariate balance before and after 1:1 propensity score matching.

Variable	Pre-Match (Salvage n = 27 vs. Primary n = 79)	Pre-Match SMD	Post-Match (Salvage n = 25 vs. Primary n = 25)	Post-Match SMD
Age		**−0.51**		**−0.12**
Male sex		+0.07		**−0.24**
BMI		**−0.15**		**+0.19**
CCI		**+0.20**		−0.03
Type II odontoid		−0.05		**−0.10**
High-energy mechanism		**+0.21**		+0.08
Propensity score		**+0.70**		+0.04

The propensity score was estimated via multivariable logistic regression on age, sex, BMI, CCI, type II odontoid fracture, and high-energy mechanism. 1:1 nearest-neighbor matching on logit (propensity score) with caliper = 0.2 × SD (7 covariates assessed). Bold dark-red standardized mean differences (|SMD| ≥ 0.10) indicate residual imbalance. The propensity score SMD decreased from +0.70 pre-match to +0.04 post-match, confirming successful matching. BMI = body mass index; CCI = Charlson Comorbidity Index; SMD = standardized mean difference.

**Table 3:** Matched outcomes: salvage versus primary C1–C2 fusion.

Outcome	Salvage (n = 25)	Primary (n = 25)	*p*-Value
***Primary Outcome***			
**LOS (days), median (IQR)**	**2.0 (2.0–3.0)**	**5.0 (3.0–6.0)**	**<0.001**
***Postoperative Radiographic Alignment***			
C2–7 lordosis (°), median (IQR)	20.8 (13.5–42.1)	33.6 (21.8–38.6)	0.101
C2–3 segmental lordosis (°), median (IQR)	17.0 (11.8–25.8)	18.5 (13.0–31.9)	0.614
C0–2 Cobb (°), median (IQR)	31.0 (20.9–40.3)	23.0 (17.4–34.9)	0.181
C2 slope (°), median (IQR)	23.6 (12.4–29.3)	13.9 (12.0–26.5)	0.277
C2 SVA (mm), median (IQR)	36.0 (23.0–47.0)	35.0 (23.5–58.0)	0.778
C1 lamina–occiput (mm), median (IQR)	10.0 (8.0–15.0)	9.0 (7.0–10.2)	0.164
***Postoperative Complications***			
Screw loosening, n (%)	0 (0.0%)	0 (0.0%)	1.000
Revision surgery, n (%)	0 (0.0%)	0 (0.0%)	1.000
Hardware failure, n (%)	0 (0.0%)	0 (0.0%)	1.000
30-day ED visit, n (%)	0 (0.0%)	2 (8.0%)	0.500
***Other***			
**Follow-up (days), median (IQR)**	**361.0 (120.0–531.0)**	**110.0 (47.0–201.0)**	**0.001**
Pedicle screw use, n (%)	11 (44.0%)	16 (64.0%)	0.164

Values are the median (IQR) or n (%). *p*-values from the Mann–Whitney U test (continuous outcomes) or McNemar test (paired binary outcomes). Bold dark-red *p*-values indicate statistical significance (*p* < 0.05). Salvage patients had significantly shorter length of stay (2 vs. 5 days, *p* < 0.001) and longer radiographic follow-up (361 vs. 110 days, *p* = 0.001). Postoperative complications were uncommon and did not differ significantly between matched groups. LOS = length of stay; SVA = sagittal vertical axis.

**Table 4:** Doubly robust average treatment effects (full-cohort AIPW analysis).

Outcome	ATE (Salvage − Primary)	95% CI	*p*-Value
***Primary Outcome***			
**Length of stay (days)**	**−2.41**	**−3.63 to −1.19**	**<0.001**
***Radiographic Alignment***			
**C2–7 lordosis (°)**	**−9.11**	**−16.30 to −1.91**	**0.013**
C2–3 segmental lordosis (°)	−2.42	−8.32 to +3.48	0.422
C2 SVA (mm)	−2.64	−11.07 to +5.78	0.538
**C1 lamina–occiput distance (mm)**	**+1.85**	**+0.10 to +3.60**	**0.038**
***Complications (Risk Difference)***			
Screw loosening	−1.87%	−4.98% to +1.24%	0.238
Revision surgery	−2.84%	−6.47% to +0.80%	0.126
Hardware failure	−0.94%	−2.79% to +0.91%	0.317
30-day ED visit	−1.95%	−5.75% to +1.85%	0.313

Average treatment effects (ATEs) estimated via augmented inverse probability weighting (AIPW), combining stabilized inverse probability of treatment weighting with outcome regression for doubly robust estimation. Variance estimated from the empirical variance in the influence function. Bold dark-red *p*-values indicate statistical significance (*p* < 0.05). In the full cohort, salvage fusion was associated with significantly shorter LOS (ATE −2.41 days, *p* < 0.001), reduced postoperative C2–7 lordosis (ATE −9.11°, *p* = 0.013), and greater C1 lamina–occiput distance (ATE +1.85 mm, *p* = 0.038). Complication risk differences were small and not statistically significant. AIPW = augmented inverse probability weighting; ATE = average treatment effect; CI = confidence interval; ED = emergency department; LOS = length of stay; SVA = sagittal vertical axis.

**Table 5:** E-value analysis for unmeasured confounding of the length of stay association.

Quantity	Value	Interpretation
Cohen’s d (standardized LOS effect)	0.65 (0.12 to 1.40)	Medium–large effect
Approximate risk ratio	1.81 (1.11 to 3.59)	Derived from Cohen’s d
**E-value (point estimate)**	**3.02**	Confounder association required to nullify the point estimate
**E-value (CI bound closest to null)**	**1.47**	Confounder association required to nullify the 95% CI

E-values per VanderWeele and Ding (Ann Intern Med 2017). An E-value of 3.02 indicates that an unmeasured confounder would need to be associated with both salvage treatment and length of stay by a risk ratio of at least 3.02 (above and beyond measured covariates) to fully explain the observed association. For the 95% CI bound closest to the null, the required confounder association is 1.47. Values were derived from the observed LOS effect using the approximation RR ≈ exp(0.91 × Cohen’s d). Bold dark-red values denote the principal E-value summary statistics (point estimate and CI-bound E-value) that the reader is intended to interpret as the primary indicators of robustness to unmeasured confounding. CI = confidence interval; RR = risk ratio.

**Table 6:** Descriptive risk difference analysis of postoperative complications (salvage vs. primary).

**Outcome**	**Salvage**	**Primary**	**Risk Difference (95% CI Upper)**	**Margin**	**Conclusion**
**Screw loosening**	0/27 (0.0%)	2/79 (2.5%)	−2.53% (upper: +0.93%)	+5.00%	**Non-inferior**
**Revision surgery**	0/27 (0.0%)	2/79 (2.5%)	−2.53% (upper: +0.93%)	+5.00%	**Non-inferior**
**Hardware failure**	0/27 (0.0%)	1/79 (1.3%)	−1.27% (upper: +1.20%)	+5.00%	**Non-inferior**
**30-day ED visit**	0/27 (0.0%)	2/79 (2.5%)	−2.53% (upper: +0.93%)	+5.00%	**Non-inferior**

Pre-specified non-inferiority margin of 5 percentage points on the risk difference scale (one-sided α = 0.025). Salvage C1–C2 fusion demonstrated non-inferiority regarding primary fusion for all four complication endpoints: the upper bound of the 95% confidence interval for the risk difference (salvage − primary) remained below the +5% margin in every case. Risk differences are presented in percentage points with the upper bound of the two-sided 95% Wald confidence interval, which corresponds to the upper bound of the one-sided 97.5% interval used for the non-inferiority conclusion. Green text in the Conclusion column denotes endpoints that met the pre-specified non-inferiority criterion. CI = confidence interval; ED = emergency department. Complication endpoints were analyzed descriptively because event counts were low. Risk differences are presented as salvage minus primary. These data should not be interpreted as definitive evidence of non-inferiority for rare complications.

## Data Availability

Data are available upon request from the authors.

## Abbreviations

The following abbreviations are used in this manuscript:

SVA	Sagittal Vertical Axis
ATE	Average Treatment Effect
IQR	Interquartile Range
BMI	Body Mass Index
CCI	Charleson Comorbidity Index
SMD	Standardized Mean Difference
LOS	Length of Stay
AIPW	Augmented Inverse Probability Weighting
CI	Confidence Interval
